# More examples of breakdown the 1:1 partner specificity between figs and fig wasps

**DOI:** 10.1186/s40529-021-00323-8

**Published:** 2021-10-09

**Authors:** Hui Yu, Yaolin Liao, Yufen Cheng, Yongxia Jia, Stephen G. Compton

**Affiliations:** 1 Plant Resources Conservation and Sustainable Utilization, South China Botanical Garden, The Chinese Academy of Sciences, Guangzhou, 510650 China; 2 Southern Marine Science and Engineering Guangdong Laboratory (Guangzhou), Guangzhou, 511458 China; 3 Centre for Plant Ecology, CAS Core Botanical Gardens, Guangzhou, China; 4 School of Biology, University of Leeds, Leeds, LS2 9JT UK

**Keywords:** Agaonidae, Co-speciation, *Ficus*, Fig wasps, Host specificity, Hybrids

## Abstract

**Background:**

The obligate mutualism between fig trees (*Ficus*, Moraceae) and pollinating fig wasps (Agaonidae) is a model system for studying co-evolution due to its perceived extreme specificity, but recent studies have reported a number of examples of trees pollinated by more than one fig wasp or sharing pollinators with other trees. This will make the potential of pollen flow between species and hybridization more likely though only few fig hybrids in nature have been found. We reared pollinator fig wasps from figs of 13 Chinese fig tree species and established their identity using genetic methods in order to investigate the extent to which they were supporting more than one species of pollinator (co-pollinator).

**Results:**

Our results showed (1) pollinator sharing was frequent among closely-related dioecious species (where pollinator offspring and seeds develop on different trees); (2) that where two pollinator species were developing in figs of one host species there was usually one fig wasp with prominent rate than the other. An exception was *F. triloba*, where its two pollinators were equally abundant; (3) the extent of co-pollinator within one fig species is related to the dispersal ability of them which is stronger in dioecious figs, especially in small species.

**Conclusions:**

Our results gave more examples to the breakdown of extreme specificity, which suggest that host expansion events where pollinators reproduce in figs other than those of their usual hosts are not uncommon among fig wasps associated with dioecious hosts. Because closely related trees typically have closely related pollinators that have a very similar appearance, the extent of pollinator-sharing has probably been underestimated. Any pollinators that enter female figs carrying heterospecific pollen could potentially generate hybrid seed, and the extent of hybridization and its significance may also have been underestimated.

**Supplementary Information:**

The online version contains supplementary material available at 10.1186/s40529-021-00323-8.

## Background

*Ficus* (Moraceae) is one of the most species-rich genera of woody plants in tropical and subtropical regions of the world (Harrison 2005), with more than 800 described species of free-standing trees, shrubs, climbers, and (hemi-)epiphytes (Corner 1965; Berg 1989, 1990). Figs are defined by their unique enclosed inflorescences (the fig or syconium) and their associated pollination system which requires entry into figs by highly-specialized fig wasps (Hymenoptera, Agaonidae). Pollinator fig wasps enter the figs to lay their eggs inside the ovules of the tiny flowers they contain. For a long period it was believed that each species of fig tree supported its own unique species of pollinator fig wasp, which was associated with no other *Ficus* species (Ramírez 1970; Wiebes 1979). When atypical pollinators were detected within figs it was assumed that these were rare mistakes that resulted in the death of the pollinators without the production of their offspring or generation of fertile seeds (Compton 1990; Ware and Compton 1992) though there was an exception of hybrids by artificial pollination (Condit 1950). More recently, it has been realized that more than one species of pollinator may be associated routinely with a single species of *Ficus*, and that widespread fig tree species can support multiple pollinators in different places but with co-occurrence in contact zones (Sun et al. 2011; Bain et al. 2016; Chen et al. 2012; Yu et al. 2019), or replace each other but with more extended overlap of distributions (Rodriguez et al. 2017), or with 2 or more pollinators over substantial parts of the range Molbo et al. 2003; Haine et al. 2006; Darwell et al. 2014). There are also examples of pollinator sharing, where two or more *Ficus* species are routinely hosts for a single species of fig wasp (Lopez-Vaamonde et al. 2001; Cornille et al. 2012; Wachi et al. 2016; Wang et al. 2016). The one-to-one relationship that was originally envisaged is now realized to have been the result of the small number of host records available from each *Ficus* species, and their limited geographical coverage within the plants’ distributions, together with the close morphological similarities of closely-related pollinators making their identification difficult. Where two or more pollinators have been recorded as the routine pollinators of a single *Ficus* species they appear to be associated with same habitat or even same tree and same syconia (Kerdelhué 1997; Haine et al. 2006; Darwell et al. 2014) though usually with a prominent pollinator (Moe et al. 2011), or different habitats (Michaloud et al. 1996), or have allopatric or parapatric distributions within the ranges of their hosts (Chen et al. 2012; Bain et al. 2016; Yu et al. 2019). However, sampling intensity is again rarely sufficient to confirm this pattern of a single species of pollinator routinely servicing each *Ficus* species at any given location.

The assumption of extreme host specificity in fig wasps was based on a combination of the host records available and the apparent specialized co-adaptations required for a fig wasp to reproduce inside the figs of each *Ficus* species. Host choice by pollinators is made by the adult females and centers on long-distance plant-specific and developmental stage-specific volatile cues released by the figs when they are ready to be pollinated (van Noort et al. 1989; Grison-Pigé et al. 2002; Hossaert-Mckey et al. 2010). Pollinator females that arrive at a fig then need to be able to negotiate their way through a narrow ostiole in order to reach the flowers where they lay their eggs, and pollinator head shape is linked to the size of the ostiole (van Noort and Compton 1996). Successful oviposition once inside a fig depends on the fig wasp having an ovipositor that is longer than the styles through which its eggs are inserted (Nefdt and Compton 1996). Finally, successful development of their offspring depends on a galling response by the plant and gall forming insects are typically highly host specific (Weiblen 2004; Yu and Compton 2012; Ghana et al. 2015; Stone and Cook 1998).

Although the relationship between fig trees and their pollinators is routinely described as a mutualism, the majority of *Ficus* species in Asia have a dioecious breeding system, where individual trees have figs that either produce only seeds (on ‘female’ trees) or only pollinator offspring (on ‘male’ trees) (Janzen 1979; Berg 2003). This situation contrasts with fig trees with a monoecious breeding system, where all the trees have figs that can produce both seeds and support the development of pollinator offspring. Monoecious fig trees are often large free-standing trees or stranglers (hemi-epiphytes) growing at low densities in forest habitats, whereas dioecious species are typically smaller and shrubby and more likely to have aggregated distributions (Berg 1990; Yang et al. 2015). Probably reflecting these differences or different wasp ecology, some pollinators of monoecious species fly and transport pollen for long distances between trees (Nason et al. 1998; Ahmed et al. 2009) increasing the possibility of host-shift by mistakes especially in Africa and America where monoecious are figs rich and be well studied (Kerdelhué et al. 1999; Machado et al. 2001; Molbo et al. 2003; Marussich and Machado 2007; Jousselin et al. 2008; Su et al. 2008; Compton et al. 2009; McLeish and van Noort 2012; Yang et al. 2015), whereas the pollinators of dioecious *Ficus* species are believed to usually display more limited dispersal (Harrison and Rasplus 2006; Chen et al. 2011; Nazareno et al. 2013) which may improve the speciation by duplication across broad geographical distributions and all the co-pollinator within one host species are sisters (Yang et al. 2015; Rodriguez et al. 2017; Yu et al. 2019). Host-shift requires coexistence of fig species within the flight range of pollinators, and is affected by flowering phenology, growing density and odor similarity between the figs. When two fig species share pollinator, they usually produce the same receptive fig odor (Cornille et al. 2012; Wang et al. 2016). Even host shifts are easy to judge among unrelated fig species, but they are likely to be easier between closely related species (Rasplus 1996) and there are numerous closely-related dioecious fig trees in Asia. Fig trees planted outside their normal range may also be more likely to support multiple pollinators, if their routine pollinators are absent locally (Corner 1965; Compton 1990; Patel et al. 1993).

The extent to which fig tree species growing within a single location are supporting more than one species of pollinator can be 20 % for both monoecious figs and dioecious figs in in southwest China (Yang et al. 2015), while that of the dioecious figs in New Guinea is only 1.5 % (Moe et al. 2011). Most recent studies have concentrated on the pollination biology of individual species of fig trees (Chen et al. 2012; Darwell et al. 2014; Bain et al. 2016; Rodriguez et al. 2017; Yu et al. 2019), though pollinator sharing resulting in gene flow between closely-related *Ficus* species has nonetheless been detected (Machado et al. 2011; Wang et al. 2016). Here, we describe a *Ficus* community approach, where figs from southern China were screened for pollinator identity. The communities included mixtures of native and planted species and trees with both monoecious and dioecious breeding systems. We address the following questions (1) is there any difference on the extent of pollinator sharing between monoecious and dioecious *Ficus*? and (2) where two pollinators are present, does one species with predominate rate (> 85 %)? (3) is there any difference on co-pollinator across geographical distribution between monoecious and dioecious *Ficus*?

## Methods

### Study site

Fig trees were sampled mainly at two sites in Guangdong province of SE China separated by about 200 km: the South China Botanical Garden (N 23°10′46″, E113°21′06″; SCBG) with an area of 333 hectares and Dinghu Mountain (N23°09′21″, E112°30′39″; DHS) with an area of 1,133 hectares. Edaphic and climatic conditions at the two sites are similar and have a subtropical monsoon climate with distinct dry and wet seasons. The dry season runs from October to March, with 80 % of annual precipitation concentrated in April–September. The mean annual temperature is 21.8 °C in SCBG (Yu et al. 2006) and 21.9 °C in DHS (Han et al. 2019), and the coldest mean monthly temperatures (13.1 °C in SCBG and 12.6 °C in DHS) occur in January.

More than 13,000 kinds of living tropic and subtropic plants including at least 15 fig species are preserved in SCBG. The fig trees that support pollinators at SCBG include five monoecious figs, *F. microcarpa*, *F. benjamina*, *F. subpisocarpa*, *F. virens*, *F. altissima*, and seven dioecious fig trees, *F. hirta*, *F. triloba* (one tree), *F. auriculata*, *F. oligodon* (two small trees), *F. hispida*, *F. variegata* var. *chlorocarpa* and *F. pumila*. In DHS, the natural vegetation comprises mainly southern subtropical monsoon evergreen broadleaved forests, reflecting moist local climatic conditions. The *Ficus* with pollinators present at DHS are *F. microcarpa*, *F. benjamina*, *F. subpisocarpa*, *F. hirta*, *F. triloba*, *F. hispida*, *F. fistulosa*, *F. variegata* var. *chlorocarpa*, *F. oligodon*, *F. erecta* var. *beecheyana* and *F. pyriformis*. The dioecious *F. variolosa* which is naturally distributed in SE China is also reported to be found in DHS, but we don’t know if there are pollinators in their syconia. The *F. auriculata* in SCBG are planted though the species is naturally distributed locally. The other dioecious *Ficus* at the two sites had not been planted, whereas the monoecious species had been planted. In the surrounding area of SCBG and DHS, some monoecious fig species, such as *F. microcarpa*, *F. benjamina*, *F. subpisocarpa*, *F. virens* and *F. altissima*, are often planted as street trees or ornamental trees.

Three closely-related dioecious fig tree species have been recorded as sharing a single species of pollinator (Wiebes 1993) and all of them distribute in DHS. We only collected pollinators of *F. pyriformis* in DHS and difficult to check pollinator sharing among them. So the sampling area was extended to nearby within Guangdong Province for *F. erecta* var. *beecheyana* (Conghua E 113°57’9″; N 23°44’58”), *F. pyriformis* (DHS and Huizhou E 115°14′49″; N 23°5′49″ and Yangchun E111°47′9″; N22°10′23″) and *F. variolosa* (DHS and Huizhou E 115°14′49″; N 23°5′49″ and Yangchun E111°47′9″; N22°10′23″). Three species were naturally-established but with a little differences in habitats: *F. erecta* var. *beecheyana* usually occur in forests or along roadsides and streams; *F. variolosa* is usually in the forest and wet areas; while *F. pyriformis* is mainly found along streams (Zhou and Gilbert 2003; Tzeng et al. 2006).

### The study species

In total, the study species include the pollinators of four monoecious and six dioecious fig species. The four monoecious species are big trees with crops of more than ten thousand figs. *F. microcarpa* and *F. benjamina* belong to subgenus *Urostigma* section *Urostigma* subsection *Conosycea*, while *F. subpisocarpa* and *F. virens* belong to the subsection *Urostigma*. Among the dioecious species, *F. hispida* and *F. fistulosa* are small trees belonging to subgenus *Sycomorus* section *Sycocarpus* subsection *Sycocarpus* (Cruaud et al. 2012), while *F. oligodon* and *F. auriculata* are two closely related small trees belonging to section *Sycomorus* subsection *Neomorphe* (Berg 2004). *F. hirta* and *F. triloba* belong subgenus *Ficus* subsection *Eriosycea*. *F. hirta* is a shrub whereas *F. triloba* is a small tree with larger crops. *F. erecta* var. *beecheyana*, *F. pyriformi* and *F. variolosa* belong subsection *Frutescentiae* are small shrubs. The monoecious species produce largely synchronous crops, and most of dioecious fig species present well defined crops even though there may be somewhat less synchronization (Yang et al. 2002; Tzeng et al. 2006). The exception is *F. hirta* which usually exhibit asynchronous within-tree fruiting with figs of different developmental stages present for longer periods on the plants (Yu et al. 2006).

### **Fig wasp DNA extraction, amplification and analysis**

The pollinators were identified using DNA sequencing (Table 1). The mitochondrial genetic marker mtCOI was sequenced from an average of 23.2 fig wasp individuals reared from male figs of each *Ficus* species (range 8–35, total 301). Five fig wasp genera were represented (Tables 1, 2). All the sequenced fig wasps were adult offspring and therefore had developed successfully in the fig tree species from which they were reared.

**Table 1:** The identities and haplotypes of pollinators reared from figs in Southern China

*Ficus* species	Location	Insects genotyped	N trees	N figs	Pollinators	Haplotypes (frequency when > 1)
*F. microcarpa*	SCBG	19	4	18	Poll. 1. *Eupristina verticillata* agg.	H1(18); H2
*F. benjamina*	SCBG	30	5	13	Poll. 1. *Eupristina* sp. 1	H1; H2(2); H3(13); H4(11); H5; H6; H7
*F. subpisocarpa*	SCBG	35	2	30	Poll. 1. *Platyscapa* cf. *hsui* sp. 1 Poll. 2. *Platyscapa* cf. *hsui* sp. 2	H1(2); H2(2); H3; H4(2); H5; H6(2); H7(8); H8; H9; H10; H11; H12; H13; H14; H15(2); H16(2); H17(2); H18(2) H1(2)
*F. virens*	SCBG	8	1	7	Poll. 1. *Platyscapa coronata*	H1(3); H2; H3(4)
*F. auriculata*	SCBG	31	1	13	Poll. 1 *Ceratosolen* cf. *emarginatus* sp. 1 Poll. 2 *Ceratosolen* cf. *emarginatus* sp. 2	H1; H2(29) H1
*F. oligodon*	DHS	29	1	8	Poll. 1 *Ceratosolen* cf. *emarginatus* sp. 2 Poll. 2 *Ceratosolen* cf. *emarginatus* sp. 1 Poll. 3 *Blastophaga* sp. 1	H1(23); H2(2); H3 H1 H1(2)
*F. hispida*	SCBG	22	9	22	Poll. 1. *Ceratosolen solmsi marchali*	H1; H2(20); H3
*F. hispida*	DHS	6	2	6	Poll. 1. *Ceratosolen solmsi marchali*	H2(5); H4
*F. fistulosa*	DHS	18	4	14	Poll. 1. *Ceratosolen hewitti*	H1(17); H2
*F. hirta*	SCBG	8	8	8	Poll. 1. *Valisia javana hilli*	H4(4); H6; H7; H8; H9
*F. hirta*	DHS	10	7	7	Poll. 1. *Valisia javana hilli*	H1(2); H2; H3(2); H4(4); H5
*F. triloba*	SCBG	1	1	1	Poll. 2. *Valisia javana hilli*	H1
*F. triloba*	DHS	21	9	17	Poll. 1. *Valisia esquirolianae* Poll. 2. *Valisia javana hilli*	H1(5); H2; H3(2); H4; H5 H1(8); H2(3)
*F. erecta var. beecheyana*	Conghua	29	6	25	Poll. 1. *Blastophaga* sp. 1	H1(8); H2(14); H3; H4; H5; H6(3); H7
*F. pyriformis*	DHS	3	1	3	Poll. 1. *Blastophaga* sp. 1	H4, H9(2)
*F. pyriformis*	Huizhou	6	1	3	Poll. 1. *Blastophaga* sp. 1	H1(2), H2, H4, H6(2)
*F. pyriformis*	Yangchun	16	1	6	Poll. 1. *Blastophaga* sp. 1 Poll. 2. *Ceratosolen* sp. 1	H2(4), H8(2), H9(9) H1
*F. variolosa*	Dangan Island	9	1	3	Poll. 1. *Blastophaga* sp. 1	H1(9)

**Table 2:** Pollinators are ranked by abundance from individual host tree species. The names of *Ficus* species are mainly from Flora of China v5 except that *F. triloba* is from the revision of Berg 2007

Subsection	Species	Trees	Location	N figs	Pollinator sp.1			Pollinator sp. 2			Pollinator sp. 3		
					Identity	N figs	N wasps (trees)	Identity	N figs	N wasps (trees)	Identity	N figs	N wasps (trees)
Monoecious													
* Conosycea*	*F. microcarpa*	3	SCBG	18	*Eupristina verticillata* agg.	18	19 (3)						
	*F. benjamina*	5	SCBG	13	*Eupristina* sp. 1	13	30 (5)						
* Urostigma*	*F. subpisocarpa*	3	SCBG	21	*Platyscapa* cf. *hsui* sp. 1	20	33 (3)	*Platyscapa* cf. *hsui* sp. 2	1	2 (1)			
	*F. virens*	1	SCBG	7	*Platyscapa coronata*	7	8 (1)						
Dioecious													
* Neomorphe*	*F. auriculata*	1	SCBG	13	*Ceratosolen* cf. *emarginatus* sp. 1	12	30 (1)	*Ceratosolen emarginatus* sp. 2	1	1 (1)			
	*F. oligodon*	1	DHS	8	*Ceratosolen* cf. *emarginatus* sp. 2	7	26(1)	*Ceratosolen emarginatus* sp. 1	1	1 (1)	*Blastophaga* sp. 1	1	2 (1)
* Sycocarpus*	*F. hispida*	2	DHS	6	*Ceratosolen solmsi marchali*	6	6 (2)						
		9	SCBG	22	*Ceratosolen solmsi marchali*	22	22 (9)						
	*F. fistulosa*	4	DHS	14	*Ceratosolen hewitti*	14	18 (4)						
* Eriosycea*	*F. hirta*	7	DHS	7	*Valisia javana hilli*	7	10 (7)						
		8	SCBG	8	*Valisia javana hilli*	8	8 (8)						
	*F. triloba*	9	DHS	17	*Valisia javana hilli*	8	11 (5)	*Valisia esquirolianae*	9	10 (4)			
		1	SCBG	1	*Valisia javana hilli*	1	1 (1)						
* Frutescentiae*	*F. erecta*	6	Chonghua	25	*Blastophaga* sp. 1	25	29 (6)						
	*F. pyriformis*	1	Yangchun	6	*Blastophaga* sp. 1	5	15 (1)	*Ceratosolen* sp. 1	1	1 (1)			
		1	Huizhou	3	*Blastophaga* sp. 1	3	6 (1)						
		1	DHS	1	*Blastophaga* sp. 1	1	3 (1)						
	*F. variolosa*	1	Dangan Island	3	*Blastophaga* sp. 1	3	9 (1)						

The names of fig wasps are mainly based on Cruaud et al. 2012. *Eupristina verticillata* is now understood to be an aggregate of similar species

*SCBG* South China Botanical Garden, *DHS* Dinghu Mountain

Genomic DNA was extracted from the whole body of each fig wasp using the EasyPure Genomic DNA Extraction Kit (TransGen, Beijing, China). A 435–710 bp fragment of the mtCOI gene for each pollinating species was then sequenced following the protocol used in previous studies (Tian et al. 2015). The reaction was optimized and programmed on a MJ Thermal Cycler (PTC 200) as one cycle of denaturation at 94 °C for 5 min, 35 cycles of 30 s denaturation at 94 °C, 30 s at a 55 °C annealing temperature, and 30 s extension at 72 °C, followed by 8 min extension at 72 °C. All amplified PCR products were purified using QIAquick spin columns (Qiagen) and were sequenced in an ABI 3730xl capillary sequencer using BigDye Terminator V 3.1 chemistry (Applied Biosystems). All unique haplotype sequences were deposited in GenBank (accession numbers: MW851213-MW851283).

We did not detect any indications of pseudo-genes, such as multiple peaks in chromatograms, stop codons or frame shift mutations (Song et al. 2008). Sequences were aligned using MUSCLE (Edgar 2004) implemented in MEGA 6.0 (Tamura et al. 2013) with manual corrections. DnaSP 5.0 was used to count the number of haplotypes (Librado and Rozas 2009). Maximum likelihood trees were constructed using Kimura-2-parameter (K2P) model by MEGA 6.0 (Tamura et al. 2013) with uniform rates for COI, and node supports were assessed based on 2000 bootstrap replicates. K2P distances within and between clades for COI haplotypes were then summarized. The clades with high gene sequence differences (larger than 0.02), were blasted to Genbank with the first 1–3 sequences sorted by percent identity. In order to determine whether pollinators collected from the same fig tree but with different geographical distribution are the same species, we further added pollinator sequences of *F. benjamina* and *F. virens* in Xishuangbanna and *F. erecta* var. *beecheyana* in Taiwan (Additional file 2: Appendix excel 1). Two species of non-pollinating fig wasps reared from *F. hirta*, *Sycoscapter hirticola* (MG548706) and *Philotrypesis josephi* (MG548673 and MG548674, both Pteromalidae) were included as outgroups (Yu et al. 2018).

## Results

### The breakdown of 1:1 specificity among sympatric monoecious and dioecious fig species

**Fig. 1:**
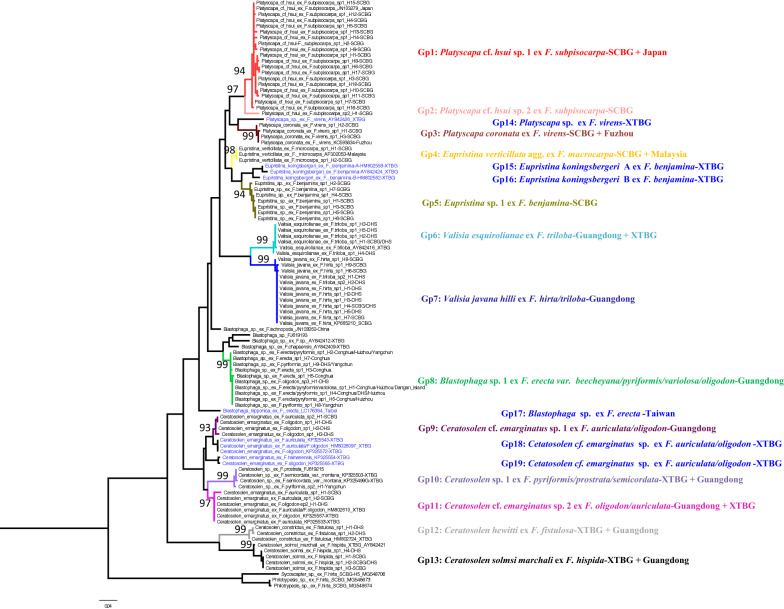
COI ML phylogenetic tree of the pollinators of the sympatric figs, with sequences of two non pollinators (Pteromalidae) as outgroups. Node support rates are shown. Haplotypes and sampling sites are also listed together with their host figs

**Fig. 2:**
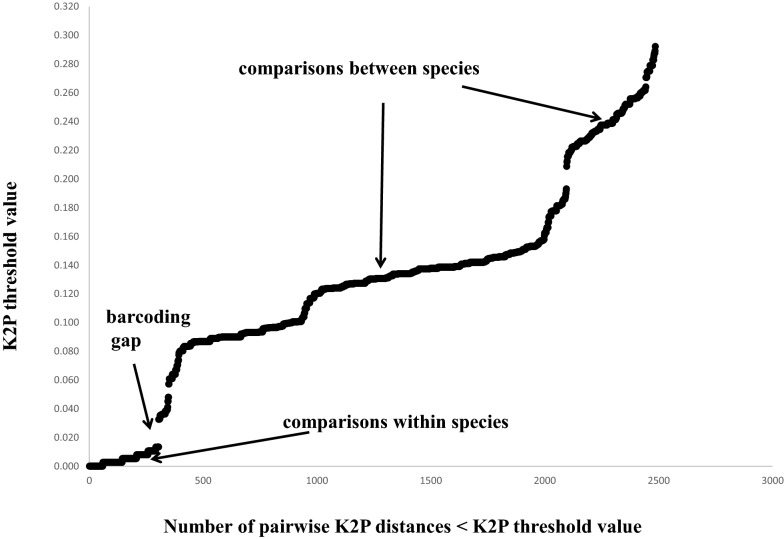
Cumulative distribution of Kimura pairwise genetic distances (K2P) for COI of pollinators associated with *Ficus* species. Intraspecific distance ranged between 0–1.3 % and interspecific distances ranged from 3.3–29.2 %. A marked barcoding gap separated the within- and between-putative species pairwise distances

Phylogenetic analyses of the COI sequences detected 13 pollinator species that had reproduced within the figs of the 13 *Ficus* species, but there was not a 1:1 concordance between them. All the pollinator clades were strongly supported (Fig. 1; Table 1), with low within-clade and large between-clade K2P distances (Additional file 1: Table S1) and the cumulative distribution of K2P distances indicating a marked barcoding gap between clades (Fig. 2). We therefore treat each clade as a distinct species. Based on the sequences downloaded from GenBank and our de novo sequencing we detected numerous examples of pollinators associated with more than one *Ficus* species and of *Ficus* species supporting the development of more than one species of pollinator. Up to three different species of pollinators were reared from the figs of a single host species and up to four host taxa were recorded for a single species of pollinator (Table 2).

The classical 1:1 pollinator and host *Ficus* relationship was only detected among two dioecious *Ficus* species (*F. hispida* and *F. fistulosa*), but it was the norm among the monoecious fig trees, where no pollinator-sharing was detected. *Ficus subpisocarpa* nonetheless supported the development of two closely-related fig wasps with 0.051 K2P distance between them, rather than one (Table 2; Fig. 1). As reported previously based on morphological identifications, the same pollinator species (*Blastophaga* sp. 1) was reared from *F. erecta* var. *beecheyana*, *F. pyriformis* and *F. variolosa*, but in addition the same species of fig wasp was also reared from figs of *F. oligodon*, an unrelated fig tree. *F. oligodon* was routinely supporting two species of *Ceratosolen*, both of which were shared with *F. auriculata* but no other species. The closely related taxa *F. hirta and F. triloba* also shared a pollinator (*Valisia javana hilli*) which was not reared from any other hosts.

**Fig. 3:**
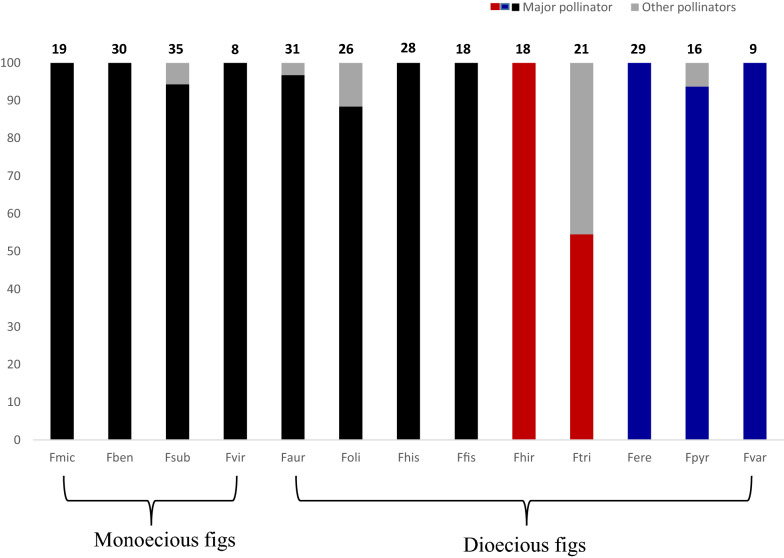
The proportion of different pollinator species in each *Ficus* species. The columns with red marks show the same major pollinator species in *F. hirta* and *F. triloba*; while those with blue marks show the same major pollinator species of *F. erecta* var. *beecheyana*, *F. pyriformis* and *F. variolosa.* The number on each column represent the sample size of each fig species within the site with more than pollinator species, the exception is *F. hirta* and *F.* *hispida* which are the total number of two sites each with only one same pollinator species

Each fig wasps were generally reared from one or two host species (Table 2; Fig. 1). *Ficus* species supporting more than one species of fig wasp generally had one predominant pollinator that provided between 88 and 97 % of the total reared individuals. The exception was *F. triloba* where its two pollinators were present in roughly equal proportions (Fig. 3; Table 1). Around half of the pollinators reared from *F. triloba* were *V. javana hilli*, a species routinely associated with *F. hirta* (*V. javana* complex sp. 1 in Yu et al. 2019).

### **Pollinator diversity in monoecious and dioecious figs with allopatric distribution**

Five cases of allopatric co-pollinator were observed, two in monoecious figs and three in dioecious figs (Fig. 1). Deep COI gene sequence divergence between subclades can be larger than 10.0 % in the pollinator of *F. virens* (Grp3 and Grp14) and *F. erecta* var. *beecheyana* (Grp8 and Grp17), or about 6 % in the pollinator of *F. benjamina* (Grp5 and Grp15 or Grp5 and Grp16) and *F. oligodon* (Grp9 and Grp19), or 1.9 % in the pollinator of both *F. auriculata* and *F. oligodon* (Grp9 and Grp18) (Additional file 1: Table S1).

At the same time, eight cases of same pollinator with broad geographical distribution were observed, three in monoecious figs and five in dioecious figs (Fig. 1). The geographical distribution of three monoecious figs with same pollinator species are different with the longest distance between SCBG and Malaysia for *F. microcarpa* (Grp4), the middle one between SCBG and Japan for *F. subpisocarpa* (Grp1), and the shortest one between SCBG and Fuzhou for *F. virens* (Grp3). While those of five dioecious figs, *F. hispida* (Grp13), *F.fistulosa* (Grp12), *F. triloba* (Grp6), *F. auriculata* and *F. oligodon* (Grp11) are all between SCBG and XTBG.

## Discussion

Our COI screening detected numerous examples of pollinator fig wasp species entering and successfully reproducing in more than a single host *Ficus* in southern China. Host overlap was frequent among pollinators of dioecious species and in most cases involved pairs of fig wasp species where one pollinator predominated and a second was reared only rarely. One interpretation of this is that the more rarely encountered pollinator species had other hosts where they were more abundant, but our screening across different *Ficus* species was not sufficiently extensive to confirm this and in some cases the pollinator species may simply be rare within our sampling area. Most examples of fig wasps developing in figs of more than one host involved fig trees that were closely related, but there were exceptions involving species of *Blastophaga* and *Ceratosolen* that were reared from figs normally associated with the other genus of pollinators though more samples need to be checked to confirm. Fig wasp offspring developing successfully in unrelated host *Ficus* has been recorded previously from Africa (van Noort et al. 2013). This ability to develop inside hosts that are phylogenetically distant shows that the host specificity of fig wasps may be determined more by the choices made by searching adult females than by any physiological limitations, and sometimes competition among fig wasps may also be involved.

Even within the *Ficus* species we sampled the size of the samples was not extensive and we are unlikely to have detected the full range of *Ficus* hosts being utilized locally by the fig wasps. There was generally one routine pollinator species combined with rarer entries by two or more additional pollinators (Moe et al. 2011; Darwell et al. 2014; Yang et al. 2015). An exception to the general pattern of pollinator sharing where one pollinator species predominant was provided by *F. triloba*, where two pollinator species were present in roughly equal numbers of its figs, but more samples, taken throughout the year, will be needed to confirm this pattern. While for its related fig, *F. hirta*, with a sampling throughout the range of the species from south China, Thailand to Indonesia, generally a single species was found, suggesting that mainly specialist pollinators in this species (Yu et al. 2019). Some of the trees we sampled were planted individuals (e.g. *F. auriculata* in SCBG) and this may have increased the extent of pollinator sharing that we detected. Our results nonetheless suggest that exceptions to the ‘classical’ one pollinator to one tree relationship are routine among sympatric dioecious fig tree species in southern China, to the extent that among trees with this breeding system strict specificity is the exception, not the norm.

Fig wasps develop inside figs on male trees of dioecious fig trees, but it is likely that similar entry by two or more pollinator species is taking place in both male and female figs. Pollinator host choice, based mainly around species-specific volatile attractants released by receptive figs, is the major isolating mechanism that helps prevent heterospecific pollen being deposited on the flowers inside female figs, but is not always effective (Souto-Vilarós et al. 2018). Other isolating mechanisms such as pollen incompatibility appear to be poorly developed in *Ficus* (Huang et al. 2019), so whenever fig wasp species are entering female figs of two more host trees in an area there is the possibility of viable hybrid seed being developed. Hybrids can mature successfully and can lead to backcrossing and introgression between species though hybrids are not common in nature (Parrish et al. 2003; Wilde et al. 2020). Some artificially generated hybrids appear to be at no reproductive disadvantage in terms of seed production, but male hybrid offspring can be sterile because pollinators cannot develop inside their figs (Ghana et al. 2015, but see also Yakushiji et al. 2012), so patterns of introgression may be complex. Meanwhile, fig species are classical plants too and the factors limiting interspecific introgression has more to do with counter-selection of hybrids than to strict pollination specificity.

Sharing of pollinators was not a feature of the monoecious fig tree species we sampled, though we still need a long-term survey of more samples. In addition, the monoecious figs in our study are mainly planted, and hence their fauna may be depauperated, which may explain fewer pollinator species in monoecious figs at some certain. Indeed, at XTBG, both *F. microcarpa* and *F. altissima* are visited by two Agaonid species, one pollinator and one cheater become from pollinator (Peng et al. 2008; Chen et al. 2013; Zhang et al. 2019, 2021). *F. benjamina* in XTBG also have two related species of pollinator (Yang et al. 2015).

Monoecious and dioecious fig trees differ in numerous ways that may influence pollinator behavior including growth form (trees versus shrubs), flowering phenology (large synchronous crops versus smaller asynchronous crops) and the generally more clumped distribution of dioecious species (monoecious species are often more dispersed). Perhaps more significantly in our study area and across SE Asia, there an exceptionally high diversity of dioecious species, most of which are pollinated by fig wasps that belong to a small number of genera. Opportunities for chance landing on figs of atypical hosts are therefore greater for those insects associated with dioecious hosts, but in addition most of the sharing of pollinators was between closely related dioecious species, which are likely to be generating relatively similar attractant cues by selection (Wei et al. 2014; Wang et al. 2016). The morphology of closely-related pollinator fig wasps is often very similar, and our results emphasize that pollinator-sharing is likely to have been under-estimated because of this. Barcoding and other molecular identification techniques are used increasingly to distinguish between fig wasp species, but our results also highlight the need to sequence fig wasps from several figs, even if they look alike, in order to detect pollinator species that may be present at low frequencies. More than one morphologically similar species can even be reproducing within the same individual figs (Sutton et al. 2017).

On the other hand, our results checked co-pollinator within one fig species across broad geographic distribution. For the monoecious figs with long dispersed pollinators, the same fig species can be pollinated by the same species of pollinator in a wide geographical range, such as *F. microcarpa* from SCBG to Malaysia, and *F. subpisocarpa* from SCBG to Japan which across a strait. The same case is in monoecious *F. racemosa* which can have same pollinator species across China-Thailand (Kobmoo et al. 2010; Bain et al. 2016). However, it is also possible for them having different pollinator species within short distance even in the same site. For example, *F. benjamina* in XTBG has two different related species (Yang et al. 2015), which may be due to niche differentiation or transferred from other related species. While for the dioecious figs, dispersing ability of the pollinators according to their ecotypes, plant size and canopy height (Yang et al. 2015) also play a certain decisive role in number of co-pollinators. As a small tree, five dioecious figs in our study can have the same pollinator species between SCBG and XTBG, although there is genetic differentiation in some of them. While for shrub species, such as *F. hirta* and *F. erecta* var *beecheyana*, they can differentiate into different species at a shorter geographical distance (Yu et al. 2019; Wachi et al. 2016).

## Conclusions

Our survey of the fig wasp pollinators associated with local assemblages of *Ficus* species in Southern China revealed contrasting pollinator relationships between monoecious and dioecious trees. Monoecious trees and their pollinators largely displayed a highly specific one pollinator for one tree association though more monoecious figs here are cultivated, and still need extensive sampling. Among dioecious species there was no such specificity, with frequent sharing of pollinators across trees and two or more species of pollinators associated with each tree species. Possible biological traits favoring this breakdown in pollinator specificity among dioecious *Ficus* include their extended asynchronous flowering phenologies and the mixtures of closely-related species that can grow in close proximity. This lack of specificity suggests that the extent of pollen flow between dioecious fig tree species is likely to have been underestimated, with unknown consequences. In addition, our results combined with other published sequences show that the dispersing distance of pollinators can determine the number of co-pollinators across a broad geographical distribution to a certain extent.

## Supplementary Information

**Additional file 1: Table S1**. COI gene sequence differences (Kimura-2-parameter) within (diagonal) and between groups (below diagonal). Within groups differences are low (highlighted in green) and are assumed to belong to the same species. Grp 1: *Platyscapa* cf. *hsui* sp. 1 ex *F. subpisocarpa*; Grp 2: *Platyscapa* cf. *hsui* sp. 2 ex *F. subpisocarpa*; Grp 3: *Platyscapa coronata* ex *F. virens*; Grp 4: *Eupristina verticillata* agg. ex *F. microcarpa*; Grp5: *Eupristina* sp. 1 ex *F. benjamina*; Grp 6: *Blastophaga* sp. 1 ex *F. erecta* var. *beecheyana/pyriformis/variolosa/oligodon*; Grp 7: *Valisia esquirolianae* ex *F. triloba*; Grp 8: *Valisia javana hilli* ex *F. hirta/triloba*; Grp 9: *Ceratosolen* sp. 1 ex *F. pyriformis/prostrata/semicordata montana*; Grp10: *Ceratosolen* cf. *emarginatus* sp.1 ex *F. auriculata/oligodon*; Grp 11: *Ceratosolen* cf. *emarginatus* sp. 2 ex *F. oligodon/auriculata*; Grp 12: *Ceratosolen hewitti* ex *F. fistulosa*; Grp13: *Ceratosolen solmsi marchali* ex *F. hispida*. Grp14-19 contain the COI sequences of pollinator from some published papers. Grp 14: *Platyscapa* sp. ex *F. virens* from XTBG (Jiang et al. 2006); Grp 15–16: *Eupristina koningsbergeri* A and B *ex F. benjamina* from XTBG (Jiang et al. 2006; Yang et al. 2015); Grp 17: *Blastophaga* sp. ex *F. erecta* from Taiwan (Wachi et al. 2016); Grp 18–19: *Cetatosolen cf. emarginatus* sp. ex *F. auriculata/oligodon* from XTBG (Wang et al. 2016).

**Additional file 2: Appendix**. Information of COI sequences used in this study. S1.1 List of pollinator sequences amplified by this study. S1.2 List of pollinator sequences downloaded from GenBank used in Fig. 1.

## Data Availability

All unique haplotype sequences of COI sequence for all the sampled pollinators were deposited in GenBank (accession numbers: MW851213-MW851283).

## Abbreviations

SCBG: South China Botanical Garden
DHS: Dinghu Mountain
XTBG: Xishuangbanna Tropical Botanical Garden
DNA: Deoxyribonucleic acid
mtCOI: Mitochondrial cytochrome oxidase I
COI: Cytochrome oxidase I
K2P: Kimura-2-parameter
