# Supplementary material for: More examples of breakdown the 1:1 partner specificity between figs and fig wasps
Source: Bot Stud. 2021 Oct 9;62:15. doi: 10.1186/s40529-021-00323-8 (PMC8502184; doi:10.1186/s40529-021-00323-8)
Supplement: Supplementary file 1 — Additional file 1: Table S1. COI gene sequence differences (Kimura-2-parameter) within (diagonal) and between groups (below diagonal). Within groups differences are low (highlighted in green) and are assumed to belong to the same species. Grp 1: Platyscapa cf. hsui sp. 1 ex F. subpisocarpa; Grp 2: Platyscapa cf. hsui sp. 2 ex F. subpisocarpa; Grp 3: Platyscapa coronata ex F. virens; Grp 4: Eupristina verticillata agg. ex F. microcarpa; Grp5: Eupristina sp. 1 ex F. benjamina; Grp 6: Blastophaga sp. 1 ex F. erecta var. beecheyana/pyriformis/variolosa/oligodon; Grp 7: Valisia esquirolianae ex F. triloba; Grp 8: Valisia javana hilli ex F. hirta/triloba; Grp 9: Ceratosolen sp. 1 ex F. pyriformis/prostrata/semicordata montana; Grp10: Ceratosolen cf. emarginatus sp.1 ex F. auriculata/oligodon; Grp 11: Ceratosolen cf. emarginatus sp. 2 ex F. oligodon/auriculata; Grp 12: Ceratosolen hewitti ex F. fistulosa; Grp13: Ceratosolen solmsi marchali ex F. hispida. Grp14-19 contain the COI sequences of pollinator from some published papers. Grp 14: Platyscapa sp. ex F. virens from XTBG (Jiang et al. 2006); Grp 15–16: Eupristina koningsbergeri A and B ex F. benjamina from XTBG (Jiang et al. 2006; Yang et al. 2015); Grp 17: Blastophaga sp. ex F. erecta from Taiwan (Wachi et al. 2016); Grp 18–19: Cetatosolen cf. emarginatus sp. ex F. auriculata/oligodon from XTBG (Wang et al. 2016). [file 40529_2021_323_MOESM1_ESM.docx]

Table S1. COI gene sequence differences (Kimura-2-parameter) within (diagonal) and between groups (below diagonal). Within groups differences are low (highlighted in green) and are assumed to belong to the same species. Grp1-13 contain the selected pollinator samples we sequenced in the study and several blasted sequences from GenBank with high percent identity. Grp 1: *Platyscapa* cf. *hsui* sp. 1 ex *F. subpisocarpa*; Grp 2: *Platyscapa* cf. *hsui* sp. 2 ex *F. subpisocarpa*; Grp 3: *Platyscapa coronata* ex *F. virens*; Grp 4: *Eupristina verticillata* agg. ex *F. microcarpa*; Grp5: *Eupristina* sp. 1 ex *F. benjamina*; Grp 6: *Blastophaga* sp. 1 ex *F. erecta* var. *beecheyana/pyriformis/variolosa/oligodon*; Grp 7: *Valisia esquirolianae* ex *F. triloba*; Grp 8: *Valisia javana hilli* ex *F. hirta/triloba*; Grp 9: *Ceratosolen* sp. 1 ex *F. pyriformis/prostrata/semicordata montana*; Grp10: *Ceratosolen* cf. *emarginatus* sp.1 ex *F. auriculata/oligodon*; Grp 11: *Ceratosolen* cf. *emarginatus* sp. 2 ex *F. oligodon/auriculata*; Grp 12: *Ceratosolen hewitti* ex *F. fistulosa*; Grp13: *Ceratosolen solmsi marchali* ex *F. hispida*. Grp14-19 contain the COI sequences of pollinator from some same fig species in our study but with different geography. Grp 14: *Platyscapa* sp. ex *F. virens* from XTBG (Jiang et al. 2006); Grp 15-16: *Eupristina koningsbergeri* A and B *ex F. benjamina* from XTBG (Jiang et al. 2006; Yang et al. 2015); Grp 17: *Blastophaga* sp. ex *F. erecta* from Taiwan (Wachi et al. 2014); Grp 18-19: *Cetatosolen cf. emarginatus* sp. ex *F. auriculata/oligodon* from XTBG (Wang et al. 2016).

|  | Grp1 | Grp2 | Grp3 | Grp4 | Grp5 | Grp6 | Grp7 | Grp8 | Grp9 | Grp10 | Grp11 | Grp12 | Grp13 | Grp14 | Grp15 | Grp16 | Grp17 | Grp18 | Grp19 |
| --- | --- | --- | --- | --- | --- | --- | --- | --- | --- | --- | --- | --- | --- | --- | --- | --- | --- | --- | --- |
| Grp1 | 0.01 |  |  |  |  |  |  |  |  |  |  |  |  |  |  |  |  |  |  |
| Grp2 | 0.051 | n/c |  |  |  |  |  |  |  |  |  |  |  |  |  |  |  |  |  |
| Grp3 | 0.124 | 0.127 | 0.006 |  |  |  |  |  |  |  |  |  |  |  |  |  |  |  |  |
| Grp4 | 0.085 | 0.107 | 0.101 | 0 |  |  |  |  |  |  |  |  |  |  |  |  |  |  |  |
| Grp5 | 0.115 | 0.129 | 0.127 | 0.05 | 0.006 |  |  |  |  |  |  |  |  |  |  |  |  |  |  |
| Grp6 | 0.164 | 0.187 | 0.184 | 0.159 | 0.157 | 0.002 |  |  |  |  |  |  |  |  |  |  |  |  |  |
| Grp7 | 0.183 | 0.196 | 0.188 | 0.164 | 0.178 | 0.12 | 0.002 |  |  |  |  |  |  |  |  |  |  |  |  |
| Grp8 | 0.118 | 0.126 | 0.152 | 0.109 | 0.127 | 0.167 | 0.166 | 0.003 |  |  |  |  |  |  |  |  |  |  |  |
| Grp9 | 0.229 | 0.241 | 0.15 | 0.168 | 0.166 | 0.202 | 0.197 | 0.157 | 0.006 |  |  |  |  |  |  |  |  |  |  |
| Grp10 | 0.178 | 0.193 | 0.185 | 0.16 | 0.167 | 0.198 | 0.202 | 0.141 | 0.093 | 0.006 |  |  |  |  |  |  |  |  |  |
| Grp11 | 0.194 | 0.214 | 0.163 | 0.158 | 0.168 | 0.186 | 0.19 | 0.178 | 0.052 | 0.081 | 0.005 |  |  |  |  |  |  |  |  |
| Grp12 | 0.353 | 0.37 | 0.283 | 0.29 | 0.314 | 0.292 | 0.291 | 0.324 | 0.281 | 0.338 | 0.286 | 0.007 |  |  |  |  |  |  |  |
| Grp13 | 0.332 | 0.381 | 0.295 | 0.283 | 0.291 | 0.313 | 0.301 | 0.301 | 0.295 | 0.313 | 0.291 | 0.152 | 0.01 |  |  |  |  |  |  |
| Grp14 | 0.105 | 0.102 | 0.101 | 0.106 | 0.111 | 0.202 | 0.187 | 0.144 | 0.211 | 0.227 | 0.214 | 0.334 | 0.328 | n/c |  |  |  |  |  |
| Grp15 | 0.107 | 0.128 | 0.131 | 0.071 | 0.068 | 0.154 | 0.175 | 0.132 | 0.189 | 0.183 | 0.174 | 0.293 | 0.314 | 0.121 | 0.011 |  |  |  |  |
| Grp16 | 0.101 | 0.12 | 0.121 | 0.069 | 0.06 | 0.169 | 0.173 | 0.131 | 0.187 | 0.156 | 0.176 | 0.306 | 0.306 | 0.119 | 0.044 | n/c |  |  |  |
| Grp17 | 0.154 | 0.155 | 0.154 | 0.111 | 0.124 | 0.196 | 0.164 | 0.126 | 0.15 | 0.156 | 0.144 | 0.286 | 0.277 | 0.169 | 0.142 | 0.146 | n/c |  |  |
| Grp18 | 0.205 | 0.216 | 0.145 | 0.15 | 0.151 | 0.178 | 0.178 | 0.139 | 0.019 | 0.091 | 0.052 | 0.274 | 0.3 | 0.187 | 0.166 | 0.173 | 0.128 | 0 |  |
| Grp19 | 0.225 | 0.238 | 0.174 | 0.157 | 0.167 | 0.204 | 0.194 | 0.18 | 0.057 | 0.095 | 0.054 | 0.293 | 0.31 | 0.228 | 0.192 | 0.192 | 0.15 | 0.053 | 0.007 |
